# Development of a risk prediction model for subsequent infection after colonization with carbapenem-resistant *Enterobacterales*: a retrospective cohort study

**DOI:** 10.1186/s13756-024-01394-5

**Published:** 2024-04-24

**Authors:** Guanhao Zheng, Jiaqi Cai, Han Deng, Haoyu Yang, Wenling Xiong, Erzhen Chen, Hao Bai, Juan He

**Affiliations:** 1 World Health Organization Collaborating Centre for Infectious Disease Epidemiology and Control, School of Public Health, Li Ka Shing Faculty of Medicine, The University of Hong Kong, Hong Kong Special Administrative Region, Hong Kong, China; 2 Department of Clinical Laboratory, Kunshan Hospital, Nanjing University of Chinese Medicine, Kunshan, China; 3 School of Medicine, Jiangsu University, Zhenjiang, China; 4 Department of International Medical Center, Shenzhen Hospital, Southern Medical University, Shenzhen, China; 5 Department of Pharmacy, Handan First Hospital, Handan, China; 6 Department of Infection Management, Chongqing University Cancer Hospital, Chongqing, China; 7 Department of Emergency Intensive Care Unit, Ruijin Hospital affiliated to Shanghai Jiao Tong University School of Medicine, Shanghai, China; 8 Department of Pharmacy, Chongqing University Cancer Hospital, Chongqing, China; 9 Department of Pharmacy, Ruijin Hospital affiliated to Shanghai Jiao Tong University School of Medicine, Shanghai, China

**Keywords:** Carbapenem-resistant *Enterobacterale*, Colonization, Infection, Prediction model, All-cause mortality, Nomogram

## Abstract

**Background:**

Colonization of carbapenem-resistant *Enterobacterale* (CRE) is considered as one of vital preconditions for infection, with corresponding high morbidity and mortality. It is important to construct a reliable prediction model for those CRE carriers with high risk of infection.

**Methods:**

A retrospective cohort study was conducted in two Chinese tertiary hospitals for patients with CRE colonization from 2011 to 2021. Univariable analysis and the Fine-Gray sub-distribution hazard model were utilized to identify potential predictors for CRE-colonized infection, while death was the competing event. A nomogram was established to predict 30-day and 60-day risk of CRE-colonized infection.

**Results:**

879 eligible patients were enrolled in our study and divided into training (*n* = 761) and validation (*n* = 118) group, respectively. There were 196 (25.8%) patients suffered from subsequent CRE infection. The median duration of subsequent infection after identification of CRE colonization was 20 (interquartile range [IQR], 14–32) days. Multisite colonization, polymicrobial colonization, catheterization and receiving albumin after colonization, concomitant respiratory diseases, receiving carbapenems and antimicrobial combination therapy before CRE colonization within 90 days were included in final model. Model discrimination and calibration were acceptable for predicting the probability of 60-day CRE-colonized infection in both training (area under the curve [AUC], 74.7) and validation dataset (AUC, 81.1). Decision-curve analysis revealed a significantly better net benefit in current model. Our prediction model is freely available online at https://ken-zheng.shinyapps.io/PredictingModelofCREcolonizedInfection/.

**Conclusions:**

Our nomogram has a good predictive performance and could contribute to early identification of CRE carriers with a high-risk of subsequent infection, although external validation would be required.

## Background

As one of the antibiotic-resistant bacteria from World Health Organization priority list, carbapenem-resistant *Enterobacterale* (CRE), which could cause a variety of intractable infections, has been regarded as a fatal medical threat with high morbidity and mortality in Chinese healthcare facilities [1–3]. CRE colonization is usually considered as a prerequisite for CRE infection [4, 5]. Among hospitalized CRE carriers, a relatively high incidence of subsequent CRE infections ranging from 8.8 to 25.5% is reported in multiple studies [6–9].

Identifying potential predictors for the switch from CRE colonization to infection is meaningful for early detecting high-risk patients and developing effectively preventive and therapeutic strategies consequently. There are multiple identified risk factors associating with CRE-colonized infection, such as prior antimicrobial exposures and comorbidities [4]. However, it is necessary to assess the impact of other potential variables like concomitant drugs utilization and evaluate the extent of importance for each significant predictive variable among hospitalized CRE carriers. Moreover, it is an urgent need for clinicians to find a convenient and precise clinical tool for evaluating the individual risk of CRE-colonized infection comprehensively. As a visualizing presentation of statistical data, nomogram is a suitable tool since it has been widely utilized to make clinical decisions by predicting the incidence, development, prognosis, and mortality of various diseases for the past few years [10–12].

Therefore, the current retrospective multicenter cohort study intended to build up a robust prediction model with nomogram for early identification of high-risk patients with CRE carriage and thus reducing the risk of CRE infection as much as possible. To this end, multiple covariates in various aspects were analyzed to screen and incorporate the independent predictors for CRE infection within 30 and 60 days after detection of CRE carriage.

## Methods

### Study design and participants

Our study was designed as a retrospective cohort investigation of the data from two tertiary hospitals in Shanghai and Chongqing city, China. It was first approved by the Institutional Review Board of the Ruijin Hospital Affiliated to Shanghai Jiao Tong University School of Medicine (2021-59; March 19, 2021), then by the Institutional Review Board of another participating center (Chongqing University Cancer Hospital). Due to the retrospective nature of our study, a waiver of written informed consent was approved in accordance with the national legislation and the institutional requirements.

Data sources were clinical charts and electronic records from individual patients in all participating hospitals, which were de-identified before analyzed by researchers. All consecutive adult patients (≥ 18 years) who admitted to the participating hospitals in general and acute medical wards from January 2011 to December 2021 with verified CRE colonization (based on microbiological culture result) were identified from our data sources and enrolled. During our research period, the prevalence of CRE colonization in general and acute medical wards from Ruijin Hospital Affiliated to Shanghai Jiao Tong University School of Medicine and Chongqing University Cancer Hospital was similarly low (0.33% and 0.18%, respectively), which was estimated by dividing the number of patients with CRE colonization by the total number of patients hospitalized in general and acute medical wards in our research period. The training cohort included all eligible patients who were hospitalized in Ruijin Hospital Affiliated to Shanghai Jiao Tong University School of Medicine, while participants from Chongqing University Cancer Hospital were distributed into the validation cohort.

Any *Enterobacterale* strain exhibiting in vitro resistance to any of the carbapenems was defined as CRE, according to the corresponding Clinical and Laboratory Standards Institute (CLSI) criteria employed in the aforesaid hospitals during the investigation period. Patients were included only once when they were detected CRE carriage for the first time. CRE colonization was defined as the isolation of CRE from rectal swab or other non-sterile samples (e.g., urine, sputum, etc.) without any clinical symptom and sign of infection. As for the procedure of CRE colonization screening, an active surveillance strategy in both hospitals was applied by weekly rectal swab sample collection during the patients’ whole hospital stay period until their confirmation of CRE carriage or discharge. For other sites, CRE colonization screening was carried out based on the local clinicians’ discretion and the policy of corresponding hospital.

### Study objectives

The measured outcome in our study was the development of subsequent CRE infection after the confirmation of CRE colonization status within 30 days (primary outcome) and 60 days (secondary outcome). Evaluation of subsequent CRE infection in different types was performed by three infectious disease specialists (two clinicians and one microbiologist) in corresponding hospital to minimize the risk of misdiagnosis, which was in accordance with the Centers for Disease Control and Prevention (CDC) criteria [13].

The severity of infection was assessed by sequential organ failure assessment (SOFA) score and the septic shock criteria, as well as Acute Physiology and Chronic Health Evaluation II (APACHE II) score for ICU patients [14, 15]. The evaluation of CRE colonization and infection were all performed by corresponding infectious disease specialists.

### Data collection for potential predictors and outcomes

Candidate prediction variables included: demographics (age, sex, weight), CRE colonization status [primary colonization organism, first specimen of colonization, number of colonization site, polymicrobial colonization, concurrent fungal colonization, time to detection of CRE colonization after admission], hepatic function [alanine transaminase (ALT), aspartate aminotransferase (AST), total bilirubin (TBil)], renal function [blood urea nitrogen (BUN), creatinine clearance (CrCl, calculated by Cockcroft-Gault formula)], invasive procedure and/or devices [Continuous Renal Replacement Therapy (CRRT), Extracorporeal Membrane Oxygenation (ECMO), mechanical ventilation, vasoactive drugs, catheterization], comorbidities, Charlson comorbidity index (CCI) score, prior healthcare history within 90 days of CRE-colonized detection [hospitalization, intensive care unit (ICU) admission, length of hospital and ICU stay, surgery, antimicrobial treatment (antimicrobial agents, combination therapy, treatment duration)], and concomitant drugs [glucocorticoids (GCs), proton-pump inhibitors (PPIs), albumin, immunosuppressants, opioids]. Concurrent fungal colonization was defined as detection of any opportunistic fungi (e.g., Candida albicans) without any clinical symptom and sign of invasive fungal infections after identification of CRE colonization, which was also assessed by three infectious disease specialists as well. The execution of fungal colonization screening depended on clinicians’ judgement [16]. The albumin supplementary therapy would be initiated when patients’ serum albumin level is lower than 3.5 g/dL. A daily dose of 300mL 20% albumin was administered until the serum albumin level rising back to 3.5 g/dL.

Clinical outcomes were also recorded, including 30-day and 60-day risk for CRE infection after colonization, time to diagnosis of CRE infection after colonization and all-cause 60-day mortality.

### Sample size

The calculation of the required sample was carried out by R (version 4.1.1) software with the pmsampsize package, according to the methods from Riley et al.’s article [17]. After our calculation, the minimum number of patients for model development (training cohort) was 799 with 200 outcomes (event rate, 0.25 by day 60) for evaluating 30 candidate predictors (6.66 events per candidate predictor) with an estimated c-statistic of 0.85 and 60-day prevalence of 0.25. According to the rules-of-thumb, a minimum sample size with at least 100 events and 100 non-events is needed for external validation of our model [18].

### Statistical analysis methods

All statistical analyses were performed by R (version 4.1.1) software with regplot, riskRegression, cmprsk, survival, ggplot2, dcurves and DynNom packages. Categorical variables were presented as numbers (n) and rates (%). Continuous variables with normal distribution or non-normal distribution were expressed as mean ± standard deviation or median and interquartile range (IQR), respectively. All tests were two-tailed and *P*-values < 0.05 were considered statistically significant.

Taking the competing risk of death into consideration, a Fine-Gray sub-distribution hazard model was chosen to investigate the correlation with predictors and cumulative incidence of CRE infection within 60 days after identifying colonization status by multivariable analysis in training cohort. If a patient was discharged (including transferred to another hospital and recovered) before 60th day after their CRE colonization, follow-up would be completed by our researchers to confirm this patient’s CRE subsequent infection and survival status for our analysis. Statistically significant variables in univariable analysis were selected as the candidate covariates into final multivariable analysis procedure. Lastly, a nomogram was developed based on the final model to predict 30-day and 60-day risk of subsequent CRE infection, while the day zero of our prediction was defined as the day of obtaining index microbiological culture of CRE.

Since CRE colonization status is a time-varying variable measured from admission till discharge, immortal time bias could occur if this is ignored, as patients with longer survival have a higher chance of CRE colonization detection [19]. Hence, to account for the potential bias, we implemented a sensitivity analysis in which we excluded patients, who died within 14 days (median time to colonization) of their admission [19, 20].

When it comes to the internal validation of the nomogram, model discrimination and calibration were assessed by using bootstrapping validation (1,000 bootstrap resamples) to avoid bias. The area under the curve (AUC) was applied for evaluating the model discrimination ability. The calibration curves were plotted to assess the consistency of model prediction. Whereafter, external validation was also adopted in the validation cohort. Decision curve analysis (DCA) was carried out to determine clinical usefulness of the nomogram by quantifying the net benefits for CRE-colonized patients in both cohorts. Lastly, we used prediction error curves (PECs) to evaluate the predictive accuracy of our model.

For benefiting for CRE-colonized patients with adhibiting our nomogram widely, we prepared an online dynamic calculator (Shiny app) which could be easily accessible by clinicians and pharmacists.

## Results

Before evaluating the predictive performance of our model, we have estimated that there were totally 17 patients with missing with missing data (13 with missing prior healthcare history, 3 with missing CCI scores, 1 with missing CRE colonization status) and 26 patients who were lost to follow-up. We finally decided to exclude patients with missing data because these patients only accounted for a small proportion (1.8%) and these data could be assumed as completely missing at random since the probability of being missing was the same for all cases. Moreover, taking the tiny proportion of loss to follow-up (2.8%) into consideration, we had also excluded these patients from present study since we assumed it would probably not interfere our result.

**Fig. 1:**
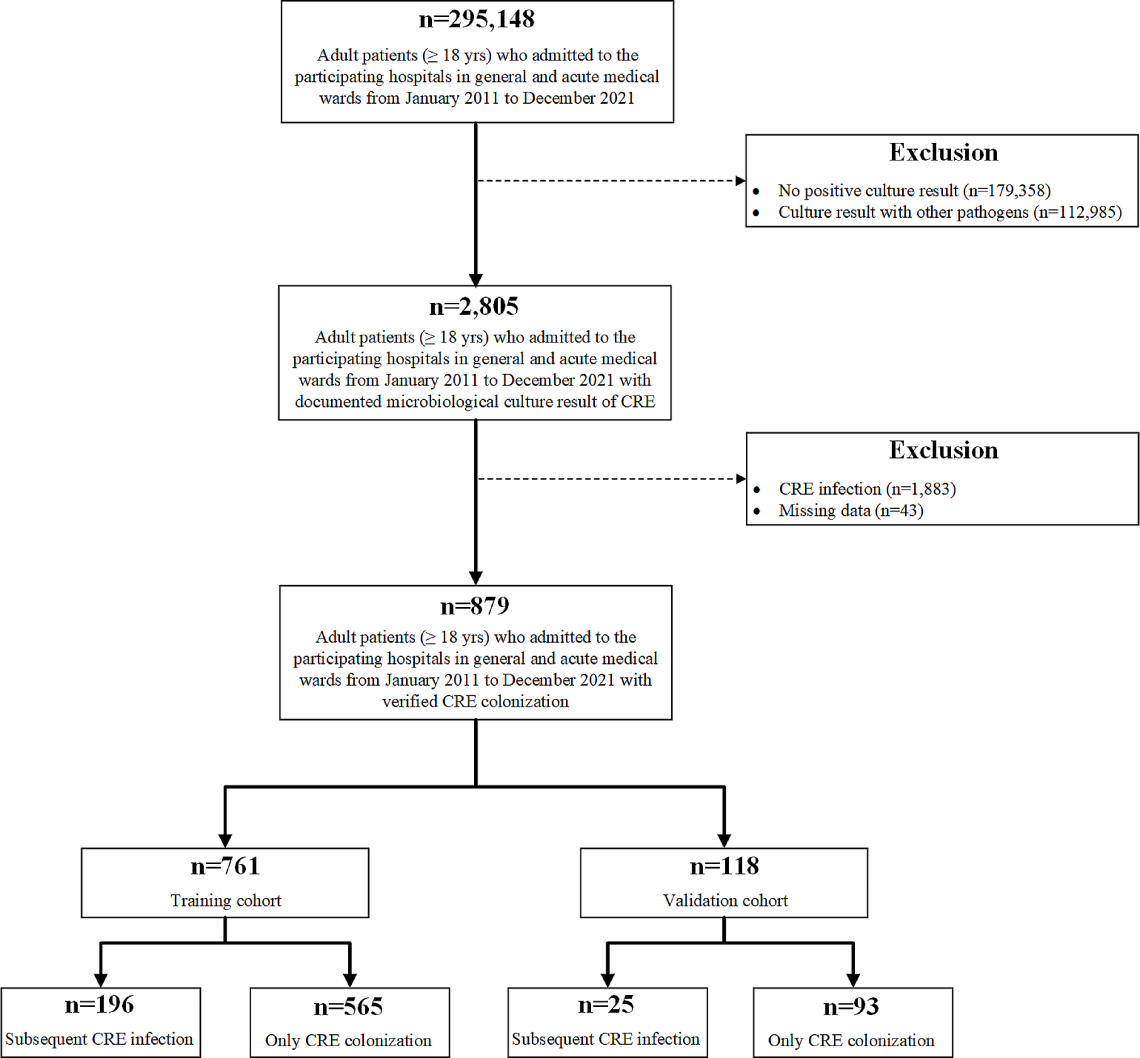
Study design

A total of 879 patients were enrolled in our final analysis during the investigation period, while 761 in training cohort and 118 in validation cohort (Fig. 1), which was nearly meeting the requirement of minimum sample size.

The characteristics of study population are listed in Table 1. The median age was 65 years and 66.6% were male. Over 90% CRE colonized strains were identified as *Klebsiella pneumoniae*. Approximately One-third and One-sixth patients were suffered from multisite CRE colonization and polymicrobial colonization, respectively. The median time to detection of CRE colonization from admission was 14 (IQR, 14–22) days. Result from sensitivity analysis exhibited that after the exclusion of patients died within 14 days of their admission, the prevalence of CRE subsequent infection within 60 days after detection of CRE colonization was similar with that from original cohorts (24.7% and 25.1%, respectively).

**Table 1:** Baseline characteristics of all patients in the training cohort and validation cohort

Variables^a^	Total *n* = 879	Training cohort *n* = 761	Validation cohort *n* = 118	P-value
**Demographics**				
Age (years)	65 (52–74)	64 (52–73)	66 (55–79)	0.026
Gender				
Male Female	585 (66.6) 294 (33.4)	513 (67.4) 248 (32.6)	72 (61.0) 46 (39.0)	0.171 -
Weight (kg)	60 (53–67)	60 (54–67)	56 (50.4–62.3)	< 0.001
**CRE Colonization status**				
Primary colonization organism				
* Klebsiella pneumoniae* * Escherichia coli*	803 (91.4) 76 (8.6)	697 (91.6) 64 (8.4)	106 (89.8) 12 (10.2)	0.527 -
First specimen of colonization				
Rectal swab Sputum Urine	573 (65.2) 183 (20.8) 123 (14.0)	496 (65.2) 164 (21.6) 101 (13.3)	77 (65.3) 19 (16.1) 22 (18.6)	0.987 0.175 0.118
Multisite colonization	294 (33.4)	257 (33.8)	37 (31.4)	0.605
Polymicrobial colonization	149 (17.0)	130 (17.1)	19 (16.1)	0.792
Concurrent fungal colonization	234 (26.6)	209 (27.5)	25 (21.2)	0.151
Time to detect CRE colonization after admission (days)	14 (14–22)	14 (14–23)	14 (14–21)	0.333
**Hepatic function**				
ALT (U/L)	44 (22–86)	46 (23–88)	35 (16.8–70.3)	0.006
AST (U/L)	39 (22–79)	39 (22–81)	33.5 (18.8–59.8)	0.021
TBil (µmol/L)	13.9 (9.5–23.1)	14.5 (10–23.6)	10 (6.4–21.5)	< 0.001
**Renal function**				
BUN (mmol/L)	5.3 (4–6.9)	5.2 (4–6.7)	6.1 (4.4–9.5)	< 0.001
CrCl (mL/min/1.73 m^2^)	69.5 (41.5–104.3)	69.0 (42.1–102.7)	71.4 (40.4–107.0)	0.952
**Invasive procedure and/or devices**				
CRRT	387 (44.0)	338 (44.4)	49 (41.5)	0.556
ECMO	14 (1.6)	13 (1.7)	1 (0.8)	0.764
Mechanical ventilation	309 (35.2)	270 (35.5)	39 (33.1)	0.607
Vasoactive drugs	256 (29.1)	207 (27.2)	43 (36.4)	0.038
Catheterization	267 (30.4)	234 (30.7)	33 (28.0)	0.541
**Comorbidities**				
Hypertension	330 (37.5)	294 (38.6)	36 (30.5)	0.090
Cardiovascular diseases	333 (37.9)	282 (37.1)	51 (43.2)	0.199
Cerebrovascular diseases	210 (23.9)	181 (23.8)	29 (24.6)	0.851
Respiratory diseases	499 (56.8)	443 (58.2)	56 (47.5)	0.028
Gastrointestinal diseases	259 (29.5)	225 (29.6)	34 (28.8)	0.867
Liver diseases	304 (34.6)	258 (33.9)	46 (39.0)	0.280
Renal diseases	291 (33.1)	257 (33.8)	34 (28.8)	0.287
Diabetes mellitus	215 (24.5)	183 (24.0)	32 (27.1)	0.470
Malignancy	187 (21.3)	157 (20.6)	30 (25.4)	0.237
Autoimmune diseases	8 (0.9)	8 (1.1)	0	< 0.001
Solid organ transplantation	22 (2.5)	22 (2.9)	0	< 0.001
**CCI score**	4 (3–5)	5 (3–5)	4 (3–5)	< 0.001
**Prior healthcare history within 90 days of CRE-colonized detection**				
Hospitalization	393 (44.7)	341 (44.8)	52 (44.1)	0.880
ICU admission	276 (31.4)	236 (31.0)	40 (33.9)	0.530
Surgery	249 (28.3)	216 (28.4)	33 (28.0)	0.925
Antimicrobial treatment	370 (42.1)	323 (42.4)	61 (51.7)	0.059
Antimicrobial agents				
CAZ/AVI Polymyxins Carbapenems Tigecycline Aminoglycosides Fosfomycin Fluoroquinolones	33 (3.8) 80 (9.1) 141 (16.0) 124 (14.1) 46 (5.2) 51 (5.8) 125 (14.2)	27 (3.5) 69 (9.1) 116 (15.2) 107 (14.1) 38 (5.0) 44 (5.8) 105 (13.8)	6 (5.1) 11 (9.3) 25 (21.2) 17 (14.4) 8 (6.8) 7 (5.9) 20 (16.9)	0.414 0.929 0.102 0.920 0.418 0.948 0.362
Antimicrobial combination therapy	140 (15.9)	115 (15.1)	25 (21.2)	0.093
Antimicrobial treatment duration (days)	25 (19–32)	26 (21–31)	19 (10–38)	0.052
**Concomitant drugs**				
GCs	382 (43.5)	333 (43.8)	49 (41.5)	0.649
PPIs	455 (51.8)	402 (52.8)	53 (44.9)	0.110
Albumin	386 (43.9)	342 (45.0)	44 (37.3)	0.119
Immunosuppressants	29 (3.3)	28 (3.7)	1 (0.8)	0.185
Opioids	98 (11.1)	91 (12.0)	7 (5.9)	0.053
**Clinical outcomes**				
CRE infection after colonization within 30 days	160 (18.2)	141 (18.5)	19 (16.1)	0.525
CRE infection after colonization within 60 days	221 (25.1)	196 (25.8)	25 (21.2)	0.287
Time to diagnosis of CRE infection after colonization (days)	20 (14–32)	21 (14.3–32)	19 (13.5–30.5)	0.443
All-cause 60-day mortality	196 (22.3)	170 (22.3)	26 (22.0)	0.941

^a^All data are exhibited as number (%) or median (IQR)

Abbreviations: ALT: alanine transaminase, AST: aspartate aminotransferase, BUN: blood urea nitrogen, CAZ/AVI: ceftazidime/avibactam, CCI: Charlson comorbidity index, CrCl: creatinine clearance, CRE: carbapenem-resistant *Enterobacterales*, CRRT: Continuous Renal Replacement Therapy, ECMO: Extracorporeal Membrane Oxygenation, GCs: glucocorticoids, ICU: intensive care unit, IQR: interquartile range, PPIs: proton-pump inhibitors, TBil: total bilirubin

The majority of patients (88.2%) in both cohorts with polymicrobial colonization were the non-fermenting bacteria carriers [mainly *Pseudomonas aeruginosa* (PA) and *Acinetobacter baumannii* (AB)]. The respiratory diseases were the comorbidities with highest incidence (56.8%) in our study, while the incidence in training cohort was higher than in validation cohort. 370 (42.1%) patients received antimicrobial treatment within 90 days before detection of CRE colonization with a median treatment duration of 25 (IQR, 19–32) days. Carbapenems, tigecycline and fluoroquinolones were the common prescribing agents. In addition, more than 40% patients received GCs, PPIs, or albumin as concomitant drug therapies during our investigation period. As for the clinical outcomes, the rate of CRE infection after colonization within 30 and 60 days were 18.2% and 25.1%, respectively. The median time to diagnosis of CRE infection after colonization was 20 (IQR, 14–32) days. All-cause 60-day mortality among carriers was as high as 22%, with similar rates between both cohorts. In general, similar baseline characteristics of patients between training and validation cohort were observed in current study, according to the data from Table 1.

**Fig. 2:**
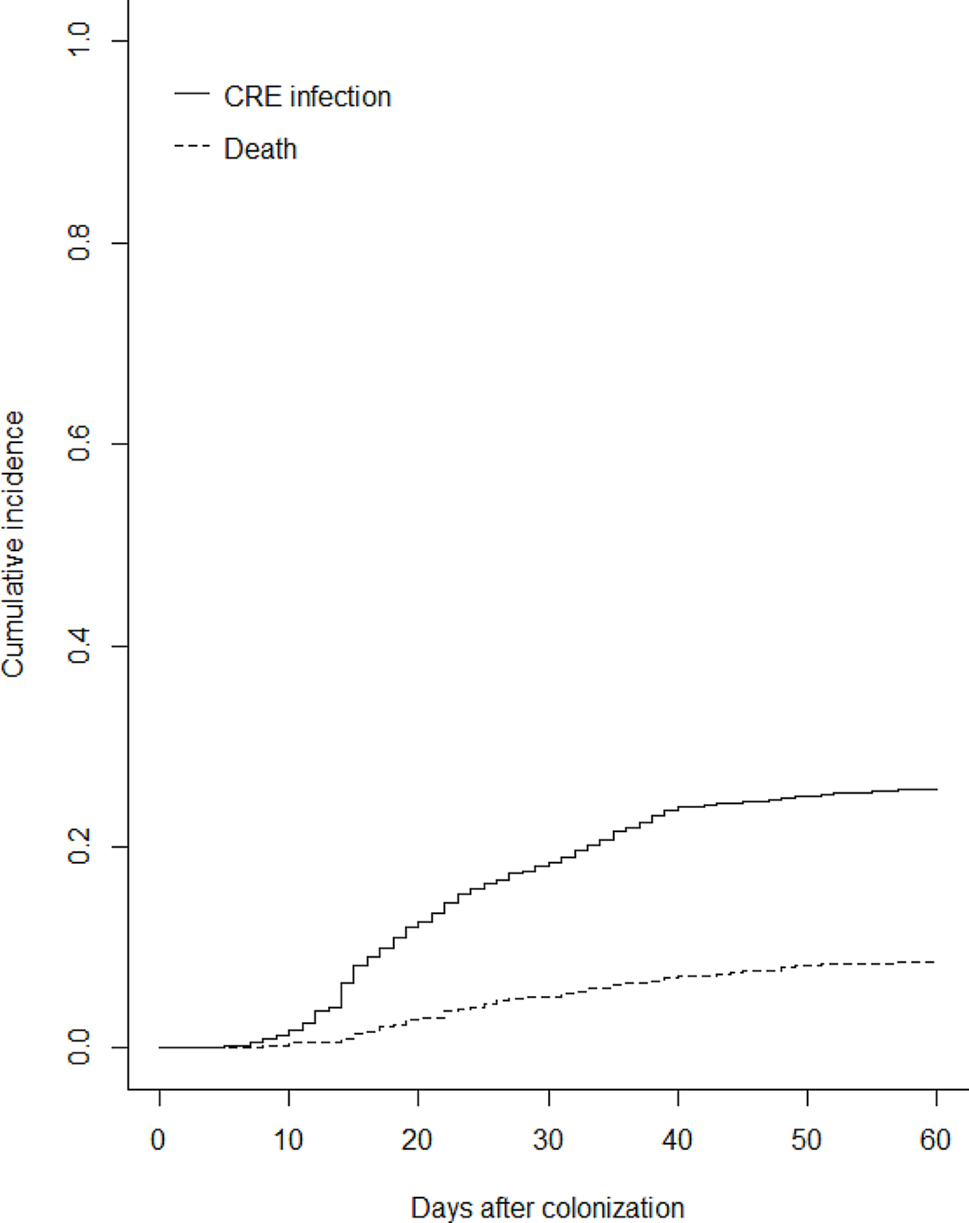
Cumulative incidence of CRE infection and death for patients with CRE colonization. A subdistribution hazard Fine-Gray model with regression was utilized to calculate the cumulative incidence. Abbreviation: CRE: carbapenem-resistant *Enterobacterales*

**Fig. 3:**
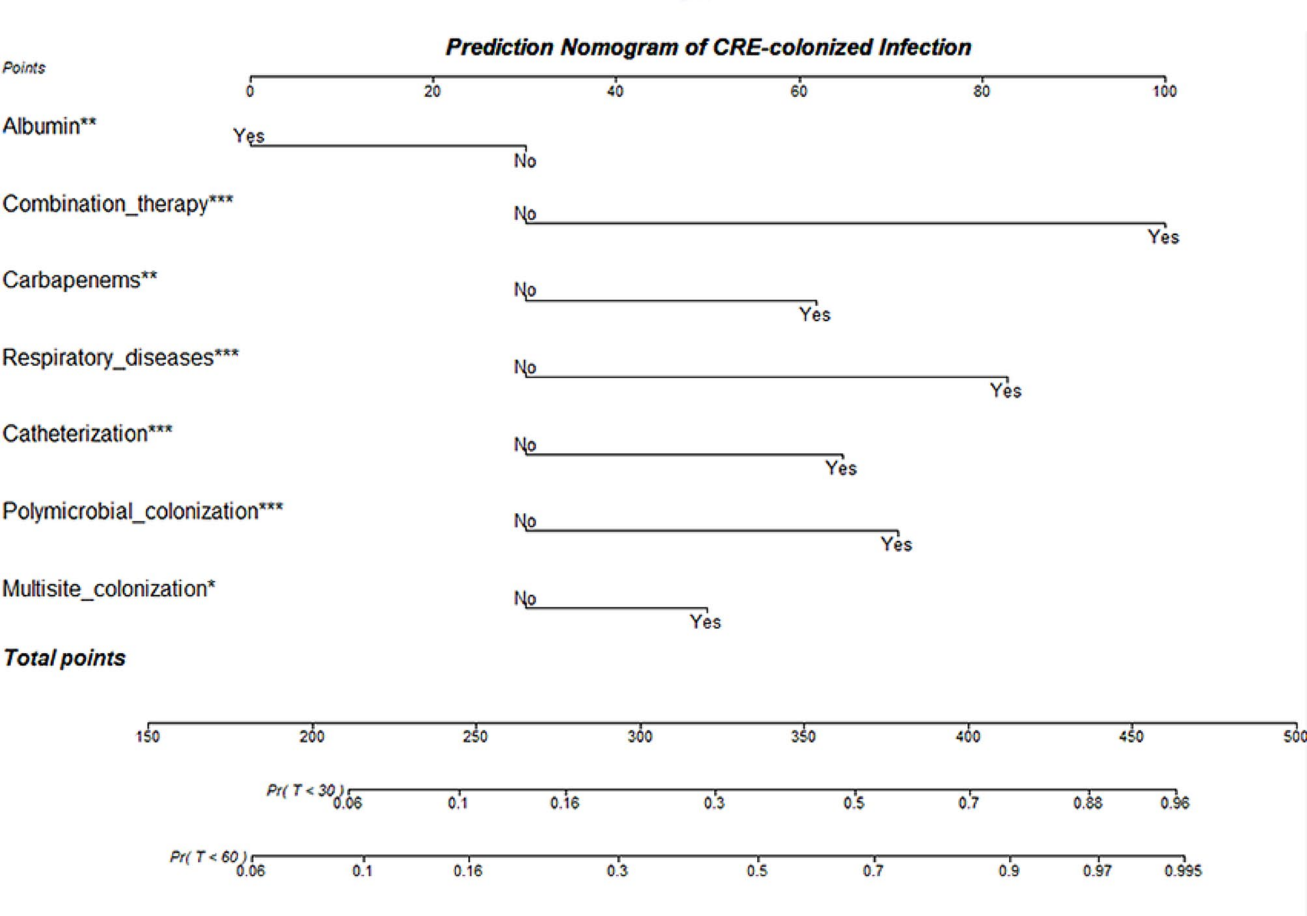
Model-informed nomogram for prediction of 30-day and 60-day cumulative risk of developing subsequent CRE infection. Abbreviation: CRE: carbapenem-resistant *Enterobacterales*; Pr: Probability; T: Time (days)

**Fig. 4:**
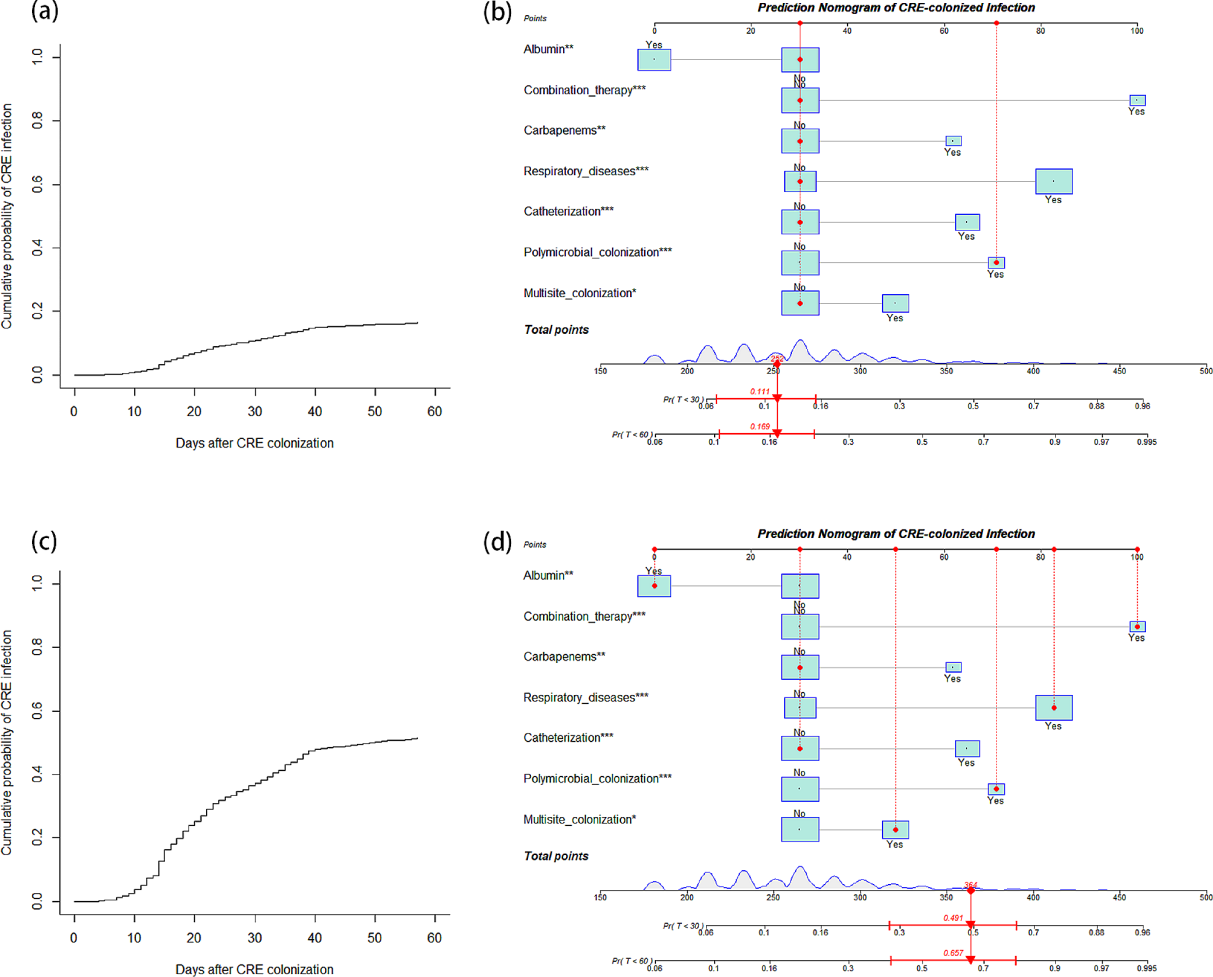
Examples of the cumulative incidence of subsequent CRE infection and nomogram-based prediction for a (a, b) low-risk and a (c, d) high-risk CRE-colonized patient. The box plot shows the categorical variables with the box size indicating percentage. The lines with gray shading on the bottom scale in the nomogram indicate the distribution of total points in the training dataset. Points on the upper scale mean ilka points for each predictor. The red arrows at the bottom of the nomogram represent the total calculated points and corresponding 30-day and 60-day predicted cumulative probability with 95% CI for the given patient. Abbreviations: CI, confidence interval; CRE: carbapenem-resistant *Enterobacterales*; Pr: Probability; T: Time (days)

The univariable analysis result was displayed in Table 2 between patients with and without subsequent CRE infection to recognize potential predictors among all candidate variables in training cohort. Figure 2 shows the cumulative incidence of CRE infection within 60 days after detection of CRE colonization and the competing event of death. Those statistically significant variables were brought into multivariable analysis with a Fine-Gray model. As a result, there were only several variables retaining in the final model, namely multisite colonization, polymicrobial colonization, catheterization and receiving albumin after colonization, concomitant respiratory diseases, receiving carbapenems and antimicrobial combination therapy before CRE colonization within 90 days (Table 3). Corresponding nomogram was established to visualize the aforementioned model as well (Fig. 3). We can easily predict the cumulative risk of CRE infection within 30 and 60 days through this useful clinical tool, as an illustration in Fig. 4. Online application of this nomogram was also presented at https://ken-zheng.shinyapps.io/PredictingModelofCREcolonizedInfection/.

**Table 2:** Comparison of patients with and without subsequent CRE infection within 60 days after detection of CRE colonization

Variables^a^	Subsequent CRE infection *n* = 196 (25.8%)	Only CRE colonization *n* = 565 (74.2%)	P-value
**Demographics**			
Age (years)	67 (57–75)	63 (49–72)	0.001
Gender			
Male Female	135 (68.9) 61 (31.1)	378 (66.9) 187 (33.1)	0.611 -
Weight (kg)	61.3 (54–69)	60 (53.8–67)	0.075
**Colonization status**			
Primary colonization organism			
* Klebsiella pneumoniae* * Escherichia coli*	176 (89.8) 20 (10.2)	521 (92.2) 44 (7.8)	0.294 **-**
First specimen of colonization			
Rectal swab Sputum Urine	131 (66.8) 34 (17.3) 31 (15.8)	365 (64.6) 130 (23.0) 70 (12.4)	0.571 0.097 0.223
Multisite colonization	80 (40.8)	177 (31.3)	0.016
Polymicrobial colonization	44 (22.4)	86 (15.2)	0.021
Concurrent fungal colonization	67 (34.2)	142 (25.1)	0.014
Time to detect CRE colonization after admission (days)	14 (14–21)	14 (14–24)	0.472
**Hepatic function**			
ALT (U/L)	49 (24–95.8)	44 (23–86.5)	0.283
AST (U/L)	44.5 (23–89)	39 (22–80)	0.260
TBil (µmol/L)	14.9 (10.5–26.2)	14.2 (9.9–22.8)	0.077
**Renal function**			
BUN (mmol/L)	5.1 (3.9–7.0)	5.2 (4.0–6.6)	0.791
CrCl (mL/min/1.73 m^2^)	54.1 (29.2–92.4)	73.4 (45.9–105.8)	< 0.001
**Invasive procedure and/or devices**			
CRRT	90 (45.9)	248 (43.9)	0.623
ECMO	6 (3.1)	7 (1.2)	0.090
Mechanical ventilation	83 (42.3)	187 (33.1)	0.013
Vasoactive drugs	62 (31.6)	145 (25.7)	0.106
Catheterization	91 (46.4)	143 (25.3)	< 0.001
**Comorbidities**			
Hypertension	81 (41.3)	213 (37.7)	0.369
Cardiovascular diseases	76 (38.8)	206 (36.5)	0.563
Cerebrovascular diseases	48 (24.5)	133 (23.5)	0.788
Respiratory diseases	150 (76.5)	293 (51.9)	< 0.001
Gastrointestinal diseases	50 (25.5)	175 (31.0)	0.149
Liver diseases	80 (40.8)	178 (31.5)	0.018
Renal diseases	82 (41.8)	175 (31.0)	0.006
Diabetes mellitus	53 (27.0)	130 (23.0)	0.255
Malignancy	48 (24.5)	109 (19.3)	0.121
Autoimmune diseases	2 (1.0)	6 (1.1)	0.961
Solid organ transplantation	7 (3.6)	15 (2.7)	0.509
**CCI score**	4 (3–5)	5 (3–5)	0.528
**Prior healthcare history within 90 days of CRE-colonized detection**			
Hospitalization	87 (44.4)	254 (45.0)	0.890
ICU admission	80 (40.8)	156 (27.6)	0.001
Surgery	64 (32.7)	152 (26.9)	0.124
Antimicrobial agents	108 (55.1)	215 (38.1)	< 0.001
CAZ/AVI PMB Carbapenems Tigecycline Aminoglycosides Fosfomycin Fluoroquinolones	9 (4.6) 31 (15.8) 59 (30.1) 30 (15.3) 25 (12.8) 14 (7.1) 36 (18.4)	18 (3.2) 38 (6.7) 57 (10.1) 77 (13.6) 13 (2.3) 30 (5.3) 69 (12.2)	0.359 < 0.001 < 0.001 0.560 < 0.001 0.343 0.031
Antimicrobial combination therapy	66 (33.7)	49 (8.7)	< 0.001
Antimicrobial treatment duration (days)	27 (21–33.8)	25 (20–30)	0.166
**Concomitant drugs**			
GCs	98 (50.0)	235 (41.6)	0.041
PPIs	117 (59.7)	285 (50.4)	0.025
Albumin	76 (38.8)	266 (47.1)	0.044
Immunosuppressants	11 (5.6)	17 (3.0)	0.095
Opioids	24 (12.2)	67 (11.9)	0.886
**Clinical outcomes**			
CRE infection after colonization within 30 days	141 (71.9)	-	-
Time to diagnosis of CRE infection after colonization (days)	21 (14.3–32)	-	-
All-cause 60-day mortality	91 (46.4)	105 (18.6)	< 0.001

^a^All data are exhibited as number (%) or median (P_25_-P_75_)

Abbreviations: ALT: alanine transaminase, AST: aspartate aminotransferase, BUN: blood urea nitrogen, CAZ/AVI: ceftazidime/avibactam, CCI: Charlson comorbidity index, CrCl: creatinine clearance, CRE: carbapenem-resistant *Enterobacterales*, CRRT: Continuous Renal Replacement Therapy, ECMO: Extracorporeal Membrane Oxygenation, GCs: glucocorticoids, ICU: intensive care unit, PMB: polymyxin B, PPIs: proton-pump inhibitors, TBil: total bilirubin

**Table 3:** Predictors of subsequent CRE infection after colonization in multivariable analysis

Variable	Subhazard ratio (95% CI)	P-value
Multisite colonization	1.39 (1.02–1.91)	0.04
Polymicrobial colonization	1.80 (1.21–2.67)	0.004
Catheterization after CRE colonization	1.69 (1.22–2.34)	0.002
Concomitant respiratory diseases	2.23 (1.56–3.17)	< 0.001
Receiving carbapenems before CRE colonization within 90 days	1.61 (1.10–2.34)	0.014
Receiving antimicrobial combination therapy before CRE colonization with 90 days	1.99 (1.28–3.11)	0.002
Receiving albumin after CRE colonization	0.65 (0.49–0.88)	0.005

Abbreviations CI: Confidence Interval, CRE: carbapenem-resistant *Enterobacterales*

**Fig. 5:**
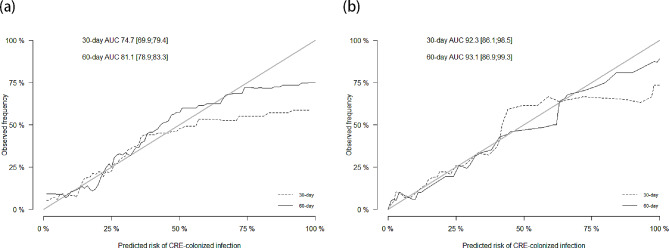
Calibration curves for the (a) training dataset and (b) validation dataset. The AUC is expressed as the point estimates and 95% CI. A clinical prediction model with an AUC value > 80 is deemed to have a good discriminatory accuracy. The 45° angle long black solid line indicates an ideal calibration, as predicted and observed probabilities are equal. Abbreviations: AUC: the area under the curve; CI: confidence interval; CRE: carbapenem-resistant *Enterobacterales*

Discrimination and calibration of our model was presented for predicting the probability of CRE-colonized infection in training cohort in Fig. 5(a), while the 30-day and 60-day AUC was 74.7 (95% CI, 69.9–79.4) and 81.1 (95% CI, 78.9–83.3), respectively. Figure 5(b) indicated that good discrimination and calibration were observed in validation cohort as well [30-day AUC 92.3 (95% CI, 86.1–98.5) and 60-day AUC 93.1 (95% CI, 86.9–99.3)].

**Fig. 6:**
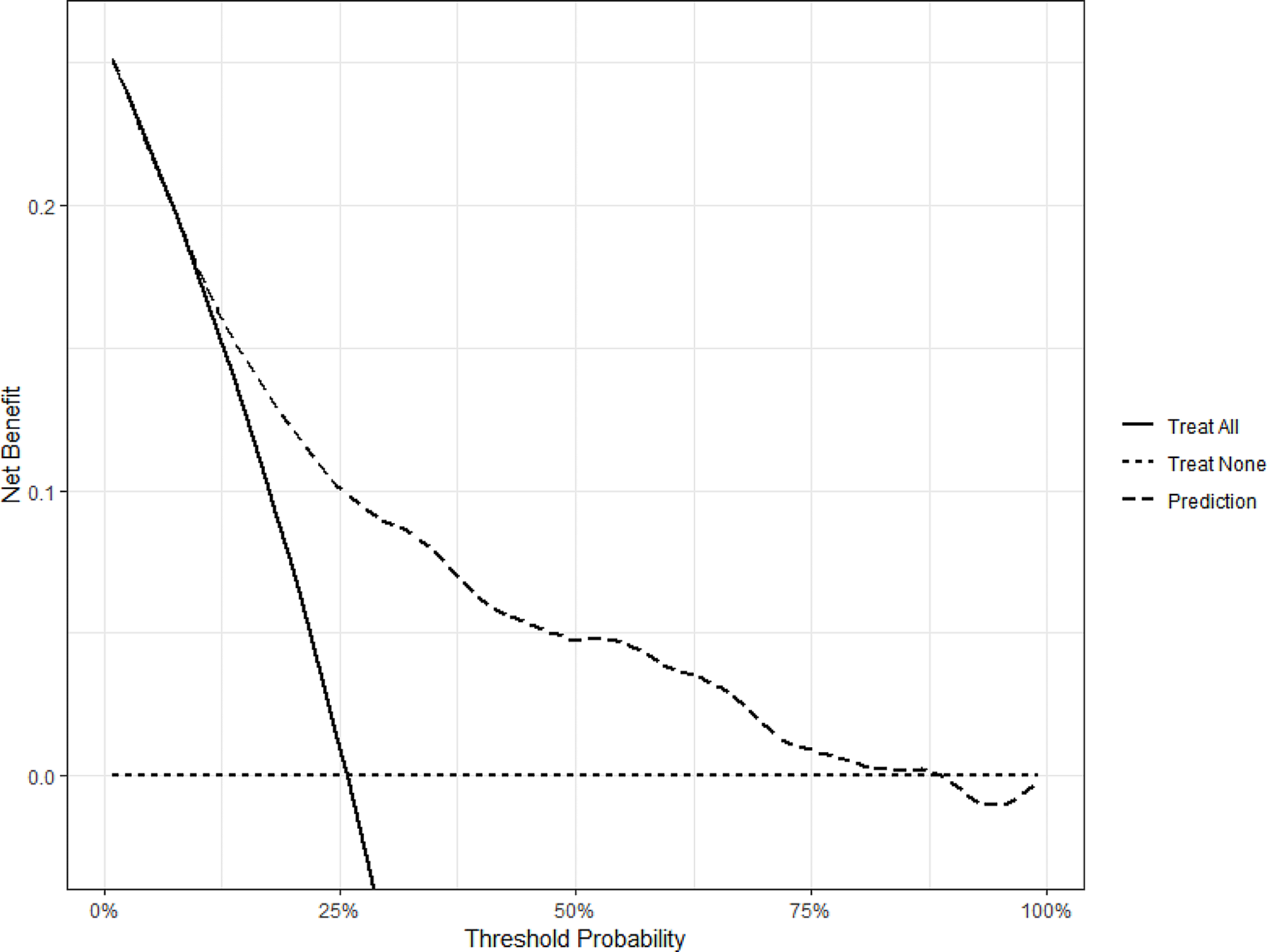
Decision-curve analysis of model-informed anti-CRE intervention. The curves represent that using the model-informed antibiotic strategy will have a higher net benefit than default strategies——“Treat All” (all patients receive active anti-CRE intervention) and “Treat None” (no patients receive active anti-CRE intervention) when CRE subsequent infection probabilities ranging from 25–88%. Abbreviation: CRE: carbapenem-resistant *Enterobacterales*

**Fig. 7:**
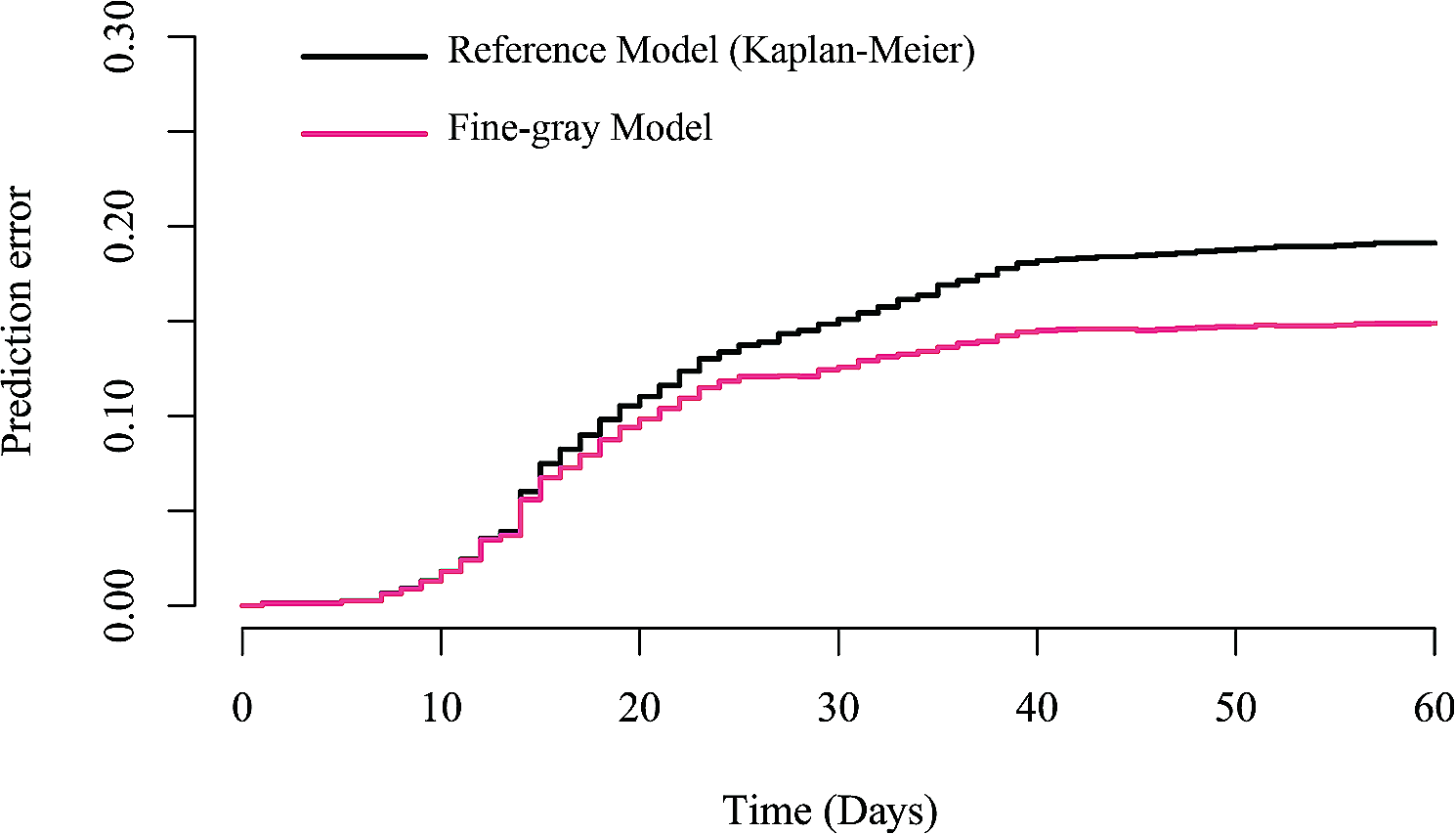
Prediction error curves for current Fine-Gray model and Kaplan-Meier (Reference) model. The curves represent that Fine-Gray model had a better prediction accuracy than referential Kaplan-Meier model, as the presence of competing risks

Ultimately, we performed DCA to evaluate the net benefit for measuring potential clinical utility of our prediction model when using model directed CRE intervention strategy in different levels of threshold probabilities (Fig. 6). The decision curve displayed that if the threshold probability of a patient or doctor is > 25% and < 88%, using the present nomogram to predict CRE infection risk adds more benefit than the treat-all-patients scheme or the treat-none scheme. The PECs indicated that our Fine-gray model had a better prediction accuracy than referential Kaplan-Meier model, as the presence of competing risks (Fig. 7).

## Discussion

To our knowledge, it is the first clinical study to investigate the potential predictors of subsequent infection after CRE colonization and constructing prediction nomogram with such a large sample size. A systematic review has demonstrated that an overall following infection probability of 16.5% was observed among CRE carriers in ten clinical studies with 1,806 patients [5]. Regarding our investigation, approximately one-fourth patients with CRE colonization suffered from subsequent infection, which had a notable influence on their 60-day survival status at the meantime, while similar result was obtained from Giannella et al.’s study [21].

We believed that our nomogram could be an effective guidance for infectious disease specialist to evaluate the individual infection risk among CRE carriers after performing AUC, calibration curves, DCA and PECs analysis through both internal and external validation. Regarding external validation, we could not neglect that the number of patients in our validation cohort is too small to meet the minimum of, which could possibly weaken the reliability of our external validation result, although the AUC value for the validation data was high. In addition, since Ramspek et al. suggested that external validation of prediction model should fully consider and interpret the competing risk to improve the reliability of model performance evaluation, we used the Fine-Gray sub-distribution hazard model and defined death as the only competing event in our study [22]. Discharge might also be considered as another potential competing event because CRE tended to be de-colonized spontaneously after patients being discharged from hospital and no longer exposed to antibiotics [23]. However, we did not adopt it because most of CRE carriers were still proved to have a prolonged colonization duration (up to 1 year) to develop CRE subsequent infection after their discharge [24–26]. Besides, we implemented follow-up for those who were discharged before 60th day after their CRE colonization to confirm if they were subsequently infected by CRE colonization and survived until the endpoint of our study.

Taking the robustness and credibility of current prediction model into account, our model is significative for early detection of high-risk CRE patients and rapid assessment for the necessity of adopting preventing or therapeutic strategies for those patients. It was reported that CRE colonization and subsequent infection might be attributed to multiple risk factors, which could be classified as four categories: patient characteristics, medical devices and operation, microbiological status, and prior antibiotic use [27]. Based upon our findings, we could summarize that all independent influence factors were included in the before-mentioned aspects, which had their own individual impacts on subsequent infection for CRE carriers.

As an invasive medical operation, indwelling catheters is thought to be an extrinsic cause of CRE colonization and infection, including central venous catheter insertion and urinary catheterization [4, 27–29]. In the process of establishing our prediction model, we had already reconfirmed that catheterization had an observably strong impact on the progress from CRE colonization to infection. However, we found that application of other invasive procedures and devices was insignificant in predicting the incidence of CRE-colonized infection, while a few papers demonstrated that using CRRT and ECMO could augment the risk of microbial colonization and infection [30–32]. This could be another worthy discussion issue to validate if CRE-colonized patients receiving CRRT or ECMO were in a high-risk status of subsequent infection.

Besides our research, multisite CRE colonization was considered as a vital influence factor in some other studies, which could probably induce subsequent CRE infection by creating a higher colonization burden [8, 21]. However, we have not evaluated multisite colonization comprehensively to figure out if there was any specific colonization site playing a predominant role in subsequent CRE infection because quite a few patients might have positive CRE colonization cultures frequently with their complex clinical conditions. What’s more, no significant difference was observed among all potential CRE colonizing sites in univariable analysis.

It is acknowledged that the pathogenesis of several infectious diseases is ascribed to polymicrobial interactions under conditions of coexistence [33, 34]. In present study, polymicrobial (mostly non-fermenting bacteria) colonization, was an independent factor affecting the development of CRE-colonized infection. Our result was consistent with the conclusion from D. Marchaim et al.’s research, which indicated that co-colonized patients with CRE and PA or AB suffered from a higher incidence rate of invasive infections and higher levels of antimicrobial resistance, as well as increasing mortality, compared with None co-colonized patients [29]. Previous studies have also reported that both PA and AB could colonize in various sites for hospitalized patients, especially in respiratory tracts for those who with lung disease [35–38], which provided us reasonable evidence for elaborating that both co-colonization and concomitant respiratory diseases were included as significant predictors in our CRE colonized-infection model.

Previous antibiotic usage before CRE colonization, including fluoroquinolones, antipseudomonal penicillins, third- or fourth-generation cephalosporins and carbapenems, was identified as an independent factor on subsequent CRE infection in various studies [4, 6, 7, 39, 40]. We have fully assessed all potentially CRE-active antibiotics and discovered that only carbapenems usage retained as a significant variable in our final prediction model, which could be a convincing impact factor on account of the satisfactory predictive performance of our model.

Furthermore, more than one-third patients receiving antimicrobial combination therapy before CRE colonization with 90 days had developed subsequent CRE infection in our study. This has been also verified as another important promoting factor on development of CRE-colonized infection for the first time. Whereas there is still no consensus on the issue if combined use of antibiotics could bring about colonization-associated infection more easily, compared with monotherapy. Opposite view was mentioned in a multicenter prospective cohort study with machine learning methods about antibiotic exposure and extended-spectrum β-lactamase-producing gram-negative bacteria (ESBL-GNB) colonization, which underscored that antimicrobial monotherapy could have a higher probability in promoting ESBL-GNB colonization and infection, compared with combination therapy [41]. It is valuable to ascertain some particular combined therapeutic schemes with potential tendency that could switch patients from CRE colonization status to subsequent infection. In addition, effective antimicrobial stewardship strategies should be implemented properly to control nosocomial CRE colonization and infection [42, 43].

As one of crucial highlights in our study, we must point out that concomitant use of albumin after CRE colonization may be a significantly protective factor on preventing patients from subsequent CRE infection, which has not been reported previously. One of possible mechanisms of using albumin in preventing patients from nosocomial colonization and infection was it could enhance the antimicrobial activity of vasostatin-I, a kind of antibacterial chromogranin-derived peptide in vivo, with its antioxidative ability [44]. Similarly, Rao et al.’s research suggested that a low serum albumin level (< 2.5 g/dl) was significantly associated with *Klebsiella*-colonized infection [45], which was a strongly support for our conclusion. With regard to that, it is still essential for confirming the exact timing of albumin supplementation for CRE-colonized patients to maximize its clinical benefit.

Good forecast performance was observed in our model to predict CRE-colonized infection, which could help us identify high-risk patients and implement suitable intervention earlier. Decolonization, which aims to rid patients of antimicrobial resistant pathogens, may be an alternative medical intervention for removing CRE strains from carriers [46]. Nevertheless, the necessity of decolonization in high-risk population should be evaluated further, since routine decolonization of CRE is not recommended due to increasing the risk of antimicrobial resistance for decolonizing agents, according to the panel consensus from European clinical guideline [47]. Our investigation provided sufficient clinical evidence for conducting decolonization with those high-risk CRE carriers precisely. In our next-step investigation, we should concentrate on verifying our conclusion in prospective studies with widely utilization of our model and finding appropriate decolonization schemes.

Our study has some limitations. First, the retrospective design of our study could possibly cause improper identification of CRE-colonized infection due to the heterogeneity in judgement with the same criteria by different specialists. In order to reduce bias, a well-designed prospective clinical study with more participants should be conducted in the future. Second, the phenotypic and genotypic detection of carbapenemases was not applied in current study, although several epidemiological studies showed that KPC-2 was the main type of carbapenemases produced in CRE strains and *bla*_KPC−2_ was the most prevalent gene in China [3, 48, 49]. It is still essential for validating if different carbapenemases types could have impact on the incidence of CRE-colonized infection. Third, although our sensitivity analysis revealed that immortal time bias could not interfere our prediction result, we should realize that this bias should be addressed in our future study since a longer immortal time tends to cause more bias and increase the magnitude of bias [50].

## Conclusion

In conclusion, about 25% patients with CRE colonization have developed subsequent infection with a negative effect on their 60-day survival status. Several significant predictors have been dug out to establish the prediction model of the probability of CRE-colonized infection. As a convenient clinical tool, our nomogram exhibits a good predictive performance, which could be useful to early identify CRE carriers with high risk of subsequent infection. It is noteworthy that concomitant utilization of albumin after CRE colonization might be an effective measure to prevent the occurrence of CRE-colonized infection. Further investigation should be carried out to validate our model and seek out appropriate preventive or therapeutic strategies for the high-risk CRE-colonized patients to lower incidence of subsequent infection and mortality.

## Data Availability

All data underlying this article will be shared on reasonable request to the corresponding author.

## Abbreviations

AB: *Acinetobacter baumannii*
ALT: alanine transaminase
APACHE II: Acute Physiology and Chronic Health Evaluation II
AST: aspartate aminotransferase
AUC: the area under the curve
BUN: blood urea nitrogen
CAZ/AVI: ceftazidime/avibactam
CCI: Charlson comorbidity index
CDC: Centers for Disease Control and Prevention
CI: Confidence Interval
CLSI: Clinical and Laboratory Standards Institute
CrCl: creatinine clearance
CRE: carbapenem-resistant *Enterobacterale*
CRRT: Continuous Renal Replacement Therapy
DCA: decision curve analysis
ECMO: Extracorporeal Membrane Oxygenation
ESBL-GNB: extended-spectrum β-lactamase-producing gram-negative bacteria
GCs: glucocorticoids
ICU: intensive care unit
IQR: interquartile range
PA: *Pseudomonas aeruginosa*
PMB: polymyxin B
PPIs: proton-pump inhibitors
Pr: Probability
SOFA: sequential organ failure assessment
T: Time
TBil: total bilirubin
